# Bioconversion of High-Calorie Potato Starch to Low-Calorie β-Glucan via 3D Printing Using *Pleurotus eryngii* Mycelia

**DOI:** 10.3390/foods11101443

**Published:** 2022-05-16

**Authors:** Hongbo Li, Suya Xie, Shangqiao Cao, Liangbin Hu, Dan Xu, Jiayi Zhang, Haizhen Mo, Zhenbin Liu

**Affiliations:** School of Food and Biological Engineering, Shaanxi University of Science and Technology, Xi’an 710021, China; hongbo715@163.com (H.L.); xiesuya111@163.com (S.X.); shangqiao@sust.edu.cn (S.C.); hulb@sust.edu.cn (L.H.); xudan@sust.edu.cn (D.X.); zhangjiayi@sust.edu.cn (J.Z.); mohz@sust.edu.cn (H.M.)

**Keywords:** bioconversion, *Pleurotus eryngii* mycelia, starch, 3D printing, β-glucan

## Abstract

Edible fungi play an important role in material and energy cycling. This study explored the role of *Pleurotus eryngii* mycelia in the transformation of potato high-calorie starch to low-calorie β-glucan. First, the 3D printing performance of the potato medium was optimized. After inoculating the fermentation broth of *Pleurotus eryngii* in 3D printing, we studied the microstructure and material composition of the product. Along with the increase in 3D printing filling ratio, the starch content of the culture product decreased from 84.18% to 60.35%, while the starch content in the solid medium prepared using the mold was 67.74%. The change in β-glucan content in cultured products was opposite to that of starch, and the content of the culture product increased from 12.57% to 24.31%, while the β-glucan content in the solid medium prepared using the mold was 22.17%. The amino acid composition and content of the 3D printing culture system and solid culture products prepared using the mold were similar. The 3D printing culture system promoted the bioconversion efficiency of mycelia. It also showed high application potential of *Pleurotus eryngii* mycelia for the preparation of low-calorie food.

## 1. Introduction

Starch is a major food reserve in plants and forms a large part of the daily calorie intake in humans. It is an important nutrient for animals, and it can have substantial positive effects on animal performance but may lead to undesirable effects on glycemic response and animal health [1,2]. Many starchy foods, especially processed foods, can be addictive and make you want to eat more than you really need [3]. Studies have reported severe gastrointestinal discomfort after the intake of denatured starch. Excess starch and sugar are easily converted into fat, especially when accompanied by high insulin levels that result from increased blood sugar levels. Starch is a main source of carbohydrate and has a high glycemic index (GI) [4]. Low GI dietary patterns result in small important improvements in established targets of glycemic control, blood lipids, adiposity, and inflammation [5]. β-glucan-enriched materials (BGEMs) were evaluated as a high-fiber and low-calorie substitute for wheat flour [6]. In addition, modification of starch promotes the reduction in calories while improving the quality of food characteristics. There is scope for the development of food products with modified low-calorie ingredients to improve the health of consumers. Starch can usually be modified to reduce digestion rate or calories through enzymatic, physical, and chemical changes in the food industry.

It is believed that limiting the hydrolysis of starch is a feasible strategy to control blood glucose levels. Adding *Hericium erinaceus* β-glucan to food can effectively reduce the level of GI, intake of blood sugar, and prevent the occurrence of diabetes [7]. Some studies have shown that β-glucan can inhibit the digestion of starch through starch protein-β-glucan complexes that encapsulate the starch and thus prevent easy access for enzymes [8]. Dextrin dextranase can convert starch or low-degree-hydrolyzed starch into glucan with 55–60% yields along with the coexistence of pullulanase or isoamylase [9]. Glucans are biologically active natural molecules and are steadily gaining attention due to their health-promoting effects, such as lowering blood sugar and lipids levels and enhancing immunity [10]. Glucans constitute the cell walls of fungi and yeast and are the main polysaccharides present in mushrooms. Most studies conducted on the health-related effects of β-glucan have characterized the modulation of the immune system, as well as metabolic and gastrointestinal effects [11]. β-glucans are polysaccharides of β-glucose monomers linked by β-glycosidic bonds. It is a type of valuable dietary fiber in cereals, mushrooms, yeasts, some bacteria, and seaweeds. Research on β-glucan has become an important branch of polysaccharide research [12]. Due to its biological activities and physiological functions, including antioxidant, hypoglycemic, anti-aging, anti-tumor, anti-viral functions, and immunity enhancement, mushrooms have been recognized as functional foods, and β-glucans are one of its components that have shown the most potential [13,14,15]. The transformation of high-calorie starch into biologically active natural glucans is a very valuable method of developing a healthy diet.

Filamentous fungi are one of the primary degraders of plant biomass because of their ability to produce enzymes that break down complex polysaccharides, including cellulose, hemicellulose, and starch [16]. Our understanding of starch degradation by filamentous fungi is mainly from studies conducted on *Aspergillus* spp. [17], which are industrially important producers of starch-degrading enzymes. Potato starch is the primary starch that is commercially used in Europe. It is generally considered that potatoes have a high glycemic index because they cause a large spike in blood sugar levels not long after eating them. Most processed potato products have a high GI, which makes potatoes not suitable for people with T2DM and obesity [18]. Edible fungi are rich in β-glucan, and we can use potato as the culture medium. We optimized the potato solid medium and attempted to use 3D printing technology to culture *Pleurotus eryngii*. Overall, this study investigated the role of *Pleurotus eryngii* mycelia in the transformation of high-calorie starch to low-calorie β-glucan during the growth of potato solid medium. Three-dimensional printing can provide a good growth environment for the growth of *Pleurotus eryngii* mycelia. Therefore, we aimed to find a novel method of accelerating the bioconversion of mycelia to high-calorie starch.

## 2. Materials and Methods

### 2.1. Chemicals and Solvents

Main materials: XG gum of a food grade was provided by Usolf Co., Ltd. (Linyi, China); potato flour and potato starch were bought from Modern Agriculture Service Co., Ltd. (Zhengyang, China); wheat bran was provided by Qianjiang Tongguang Flour Co., Ltd. (Qianjiang, China); sodium chloride, lipase, pepsin, amylase and amyloglucosidase were bought from Aladdin Biochemical Technology Co., Ltd. (Shanghai, China); 3,5-dinitrosalicylic acid, beta Dextran standard, glucose standard, and Congo red dye were provided by McLean Biochemical Technology Co., Ltd. (Shanghai, China).

Main solvents: glycerol; phosphate buffer; sodium acetate buffer; potassium chloride buffer; *Pleurotus eryngii* liquid fermentation broth: the triangular flask was filled with liquid medium, the activated slant strains were inserted, and the culture was shaken at 28 °C and at 180 r/min for 8 days.

### 2.2. Methods

#### 2.2.1. Ink Preparation

Different concentrations of food glue (XG) were mixed with potato whole flour, potato starch, wheat bran, and NaCl. The materials were evenly mixed using an electric mixer (JJ-1A digital display, Tianjin Xinbode Instrument Co., Ltd., Tianjin, China), deionized water was added (3 times of the mixed powder), and then the powder mixture was stirred using a homogenizer (IKA, T18BS25, Staufen im Breisgau, Germany) for 3 min.

#### 2.2.2. Rheology

Following the method described by Liu et al. [19], the rheological properties of the different ink samples were determined using a rheometer (AR-2000 ex, TA Co., Ltd., Kanto, Japan), using a 40 mm geometry plate with a gap of 1000 μm. Before testing, a thin film of silicone oil was applied around the trap to eliminate the effect of water evaporation. For the static shear rheological test, the shear rate was set at 0.1–10 s^−^^1^, and the viscosity and stress of the samples were measured at 25 °C. For the dynamic shear rheological measurement, which was conducted to analyze the viscoelastic properties of the samples, the temperature was set to 25 °C and the strain value was set to 0.1% (in the linear viscoelastic region), and the scanning frequency was set to 0.1–15 Hz.

#### 2.2.3. Texture

Based on the method described by Da Silva Costa et al. [20], the texture analysis of different samples was carried out using a texture analyzer (TX-XT Plus, Beijing Oriental Anno Technology Co., Ltd., Beijing, China), which was calibrated using a 1 kg weight before testing. A cylindrical probe P/75 (75 mm diameter) was used to determine the hardness, chewiness, springiness, and gumminess of the samples in full texture mode. The following experimental conditions were selected and used for all TPA trials: pre-test speed of 5.0 mm/s; test speed of 2.0 mm/s; post-test speed of 2.0 mm/s; compression of 45%, and a rest period of 5 s between the two cycles; trigger force of 10.0 g and data acquisition rate of 100 points per second. The temperature used was room temperature (25 ± 1 °C), and the measurements were taken in triplicate to obtain an estimate of the starch gel texture.

#### 2.2.4. 3D Printing and Sterile Inoculation

Three-dimensional printing: in the preliminary trials, it was observed that when the medium was inoculated into different fungal fermentation broths, it was found that the growth rate of mycelia was the fastest after inoculating the fermentation broth of *Pleurotus eryngii* for a period of time. This fermentation broth was chosen to be used in later experiments. Then, Repetier Host 2.0.0 software which was created by Marcus Littwin (Hot-World GmbH & Co. KG, Willich, Germany) was used to debug and control the extrusion of the 3D printer (CSE 1, Hangzhou Shiyin Technology Co., Ltd., Hangzhou, China). The 3D printer was set at a nozzle diameter of 1.25 mm, a layer height of 1.18 mm, a print height of 3 mm, a print speed of 30 mm/s, and the print temperature was set at 25 °C. The cylinder model was imported into the software and adjusted to the correct size. The prepared materials were filled into the 3D printing tube, and the fermentation broth was added, printed, and the printing effect was observed in the different samples.

Aseptic inoculation: The optimal medium for 3D printing was added into the vertical high-pressure steam sterilizer (SQ510C, Chongqing Yamato Technology Co., Ltd., Chongqing, China) for sterilization. Then, a certain amount of fermentation broth was added and mixed evenly to fill the 3D printing tube, and then, the rest of the 3D printing operation was conducted. The samples required were sterilized in advance, and all operations were performed at a purification workbench (SW-CJ-2F, Shaoxing Jingmai Instrument Equipment Co., Ltd., Shaoxing, China). Three groups of samples were printed, with the filling ratios set to 0%, 50%, and 100%, respectively, while the filling modes of the printed samples were set to linear and honeycomb.

#### 2.2.5. Scanning Electron Microscope

After the samples were freeze-dried, they were cut into thin slices and fixed on the electron microscope sample holder using conductive double-sided tape, and the excess powder was blown away using an ear-washing ball. After the samples were sprayed with gold on the ion coater, they were scanned and observed under a field emission scanning electron microscope (Hitachi Regulus8100, Shenzhen Keshida Electronic Technology Co., Ltd., Shaoxing, China) (5–10 kV) with an accelerating voltage magnification of 5000 times, and clear images were captured. Ten fields were observed and captured in each sample.

#### 2.2.6. Determination of Starch Content

Based on the method described by Hall et al. [21] for the optimized determination of starch content, the sample was processed as follows: 1 g of the sample was weighed, placed in a funnel along with a folded filter paper, and the fat was washed 5 times using 25 mL of ether, and then, soluble sugars were washed using about 50 mL of 85% ethanol, and the residue was transferred into a 250 mL beaker. In the beaker, the filter paper and funnel were washed with 50 mL of water, the solution was added into the beaker, and the beaker was heated in a boiling water bath for 15 min to gelatinize the starch, and then allowed to cool to below 60 °C. Then, 20 mL of amylase solution was added at 55–60 °C for 1 h and mixed constantly. Then, 1 drop of this solution was added. When 1 drop of the solution is added, it should not turn blue. If it was blue, then the gelatinization was heated again, and 20 mL of amylase solution was added and continuously kept warm until the iodine did not appear blue. Then, the mixture was heated to its boiling point, cooled, and transferred into a 250 mL volumetric flask, water was added to the mark, mixed, filtered, and discarded after the initial filtrate. Then, 50 mL of the filtrate was placed in a 250 mL Erlenmeyer flask and deionized water was added until the mark and refluxed for 1 h in a boiling water bath. Then, 2 drops of methyl red indicator solution were added after the mixture was cooled, and then the mixture was neutralized using a 20% sodium hydroxide solution. Then, the solution was transferred into a 100 mL volumetric flask, and water was added to the mark and mixed for later use.

Sample solution measurement: 5 mL of alkaline copper tartrate A solution and 5 mL of alkaline copper tartrate B solution was added into a 150 mL conical flask. Then, 10 mL of water and about 9 mL of glucose standard solution was added dropwise using a burette. Heat to was controlled to boil within 2 min and the solution was kept in a boiling state and glucose was continuously dripped into the solution at the rate of one drop every two seconds, until the blue color of the solution just about faded. The total volume of the glucose standard solution consumed was recorded and labeled as V_1_, and three replicates were performed at the same time, the average value was taken, and the amount of alkali used per 10 mL was used. The copper tartrate solution is equivalent to the mass m_1_ (mg) of glucose. The equation used was:

(1) $$X_{1}=\frac{m_{1}}{\frac{50}{250}\times \frac{V_{1}}{100}},$$

At the same time, 20 mL of water and the same amount of amylase solution as the sample solution was added, and the reagent blank solution was titrated using a glucose standard solution until the end point was reached. Then, the difference between the volume consumed and the volume of glucose standard solution consumed during calibration was calculated and was found to be equivalent to 10 mL of sample solution. The amount of glucose contained in the liquid was m_0_ (mg). The equation used was:

(2) $$X_{0}=\frac{m_{0}}{\frac{50}{250}\times \frac{10}{100}},$$

The content of starch in the sample was calculated using the following formula:

(3) $$X=\frac{\left(X_{1}-X_{0}\right)\times 0.9}{m\times 1000},$$

where m refers to the mass of the sample.

#### 2.2.7. Determination of β-Glucan Content

The colorimetric assay method for β-glucans is based on the specific interactions between Congo red dye and β-glucan and is assessed using the bathochromic shift of the visible absorption maximum of Congo Red [22]. The Congo Red method is based on the method described by Semedo et al. [23]. Drawing of the standard curve: the reaction mixture consisted of 140 μL of various concentrations of β-glucan standard solutions and 140 μL of 244 μM Congo red solution in 15 mM phosphate buffered saline at a pH of 7.2 (PBS), which were mixed in a cuvette to reach a final volume of 280 μL. The color was developed at 25 °C for 10 min. The absorbance was measured at 550 nm using a UV-Vis Spectrophotometer (TU-1950, Beijing Puyan General Instrument Co., Ltd., Beijing, China) and was compared with that of a blank reaction mixture in which the standard solutions of β-glucan were replaced with deionized water. All concentrations of the standard solutions were prepared in triplicate. The calibration curve was constructed using standard solutions of β-glucan obtained from barley and were prepared by dissolving β-glucan in 1 M NaOH, and then the solutions were neutralized using HCl. Based on the β-glucan content in the standard solution X (mg) and the absorbance value Y of the standard curve, the regression equation was obtained: Y = 0.0007X + 0.0002, R^2^ = 0.9981.

Sample determination: A blank sample was prepared by adding 140 μL of the sample to 140 μL of PBS buffer, and the measurement was carried out against 140 μL of deionized water using 140 μL of PBS. Finally, the β-glucan content in the sample was calculated using the standard curve equation.

#### 2.2.8. Determination of Amino Acid Composition and Content

Pretreatment of the hydrolyzed amino acid samples: 200 mg of the sample was weighed and added into a special hydrolysis tube (to prevent it from hanging from the wall), and 5 mL of analytically pure hydrochloric acid (about 6 mol/L) was added, the tube was sealed using an alcohol torch and placed in an oven (DHG-9030A, Henan Qiangding Machinery Equipment Co., Ltd., Hennan, China) for hydrolysis at 110 °C for 24 h. The hydrolyzed sample was taken out and cooled to room temperature. After the tube was opened, the contents were filtered into a 50 mL volumetric flask with a small funnel and filter paper. The hydrolysis tube was washed several times using deionized water, the washing liquid was filtered into a volumetric flask, the filter paper was washed with deionized water, and the volume was made into a constant to the scale line. Then, 2 mL of the sample was aspirated at a constant volume and put in the oven (DHG-9030A, Henan Qiangding Machinery Equipment Co., Ltd., Hennan, China) for deacidification. The temperature was set to 60 °C, and it was left to dry until a little solid or stain was left on the bottom for testing.

HPLC analysis of amino acids: The method described by Francioso et al. [24] was optimized. A high-performance liquid chromatograph (Agilent-1100, Shanghai Junqi Instrument Equipment Co., Ltd., Shanghai, China) was obtained for amino acid analysis, where the chromatographic column used was ODS HYPERSIL (250 mm × 4.60 mm, 5 µm); column temperature was 40 °C; mobile phase A: 6.50 g of crystalline sodium acetate was added to 1000 mL of deionized water and dissolved. Then, 200 µL of trimethylamine and 5% acetic acid were added to adjust the pH to 7.20 ± 0.05. Finally, 5 mL of tetrahydrofuran was added, the mixture was mixed and set aside; mobile phase B: 6.50 g of crystalline sodium acetate was dissolved in 200 mL of deionized water to adjust the pH to 7.20 ± 0.05 using 5% acetic acid. Then, 400 mL of acetonitrile and 400 mL of methanol were added; flow rate: 1.00 mL/min; UV detection wavelength: 338 nm, 262 nm (Pr0, Hypro); sample derivatization: 1 µL of the sample solution was added to 5 µL of boric acid buffer and mixed 3 times. Then, 1 µL of 0PA solution was added and mixed 15 times; 1 µL of FMOC-Cl solution was added and mixed 15 times; 32 µL of deionized water was directly injected into the sample after mixing it 5 times. The gradient elution procedure is shown in Table 1, and the external standard method was used for quantification.

## 3. Results

### 3.1. Rheological Test Analysis

**Figure 1:**
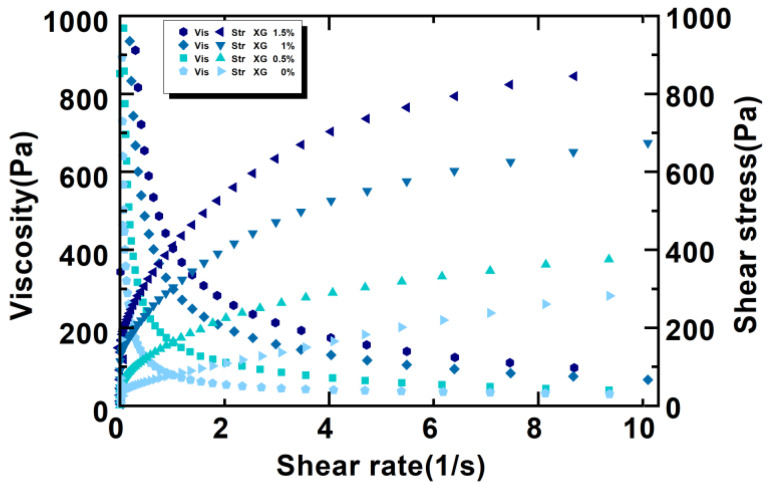
Static rheological curves of the potato gel system with different amounts of xanthan gum added.

Figure 1 shows that along with the increase in the shear rate, the viscosity of the material decreased rapidly, and then tended to be flat. The higher the shear rate, the greater the degree of viscosity reduction. When the shear rate gradually increased to around 10 s^−1^, the viscosity of the material tended to be stable. At this stage, when the shear rate increased, the viscosity of the material without xanthan gum and 0.5% xanthan gum decreased the fastest, and the material exhibited shear thinning behavior. The rapid decrease in viscosity was due to the network structure of the potato starch gel, which was disrupted by the shear rate, causing more and more starch molecules to flow instead of maintaining the network structure. As the shear force increased, the molecular chain had more breaks, and the viscosity of the entire gel system decreased at a faster rate. At the same shear rate, when more xanthan gum was added, the viscosity of the material increased further. It may have been the high viscosity of xanthan gum that made the mixture more viscous. Zhong et al. [25] found that static viscosity and shear-thinning behavior of the xanthan solutions were related but not proportional to the xanthan concentration over a wide range. Dintzis et al. [26] reported that the data showed a complex correlation between xanthan gum concentration and viscosity. The increase in viscosity shown along with the increase in the concentration of xanthan gum was attributed to intermolecular interactions or entanglements that increased the effective macromolecular size and molecular weight. In addition, Chen et al. [27] also reported similar results in the starch–polysaccharide system.

As the shear rate increased, the shear stress first increased correspondingly and then tended to level off. During the early stage of shearing, a large shearing stress was required to destroy the network structure of the entire material. Thereafter, there was an increase in the number of starch molecules, and the whole potato flour broke from the initial network structure and showed directional flow, indicating a “liquid-like” behavior, which resulted in the leveling off of shear stress.

**Figure 2:**
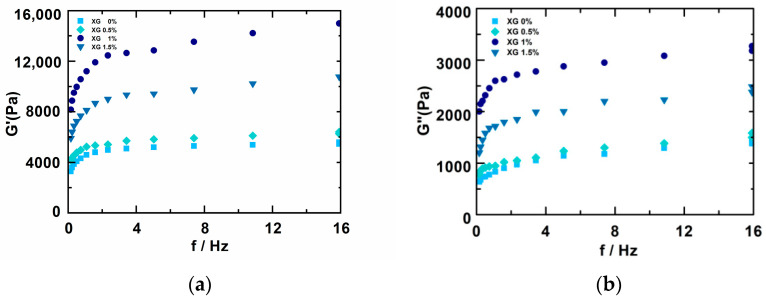
Effect of the addition of different amounts of xanthan gum on the viscoelasticity of potato starch gel. (**a**) Changes in G’ in the samples; (**b**) Changes in G” in the samples.

As shown in Figure 2, the change in G’ and G” of the different materials were similar, after adding different proportions of xanthan gum. G’ was always greater than G”, indicating that the sample had a highly elastic gel structure. As the frequency and the amount of xanthan gum added increased, the G’ and G” values showed a gradually increasing trend when low levels (0%, 0.5%) of xanthan gum were added, which may have been due to new hydrogen bonds being created between xanthan gum and potato starch, and they increased the viscoelasticity of the whole system. However, when a higher amount of xanthan gum (1.5%) was added, G’ and G” did not continue to increase at a similar rate, and G’ and G” were slightly lower than when 1% xanthan gum was added, which meant that a higher content of xanthan gum had a negative effect on the viscoelasticity of the entire material system, and that a high content of xanthan gum competed with potato starch for water, thereby interfering with the formation of the starch three-dimensional network structure [28]. Wang et al. [29] and Ronda et al. [30] also reported similar results after the addition of hydrocolloids.

### 3.2. Texture Profile Analysis

The TPA analysis (Table 2) showed that the addition of a small amount of xanthan gum first decreased and then increased the hardness of the material, and this may have been due to the good hydrophilicity and stability of the xanthan gum. The addition of xantham gum to the material gave the material the ability to maintain good water retention capacity and form a weaker gel structure, which reduced the hardness of the material. When the amount of xanthan gum added reached 1.5%, the hardness of the material increased again, which may have been because the excess xanthan gum may have made the network structure stronger and improved the overall rigidity of the material. As the amount of xanthan gum increased, the elasticity, chewiness, adhesiveness, and resilience of the material all increased at first and then decreased. When the amount of xanthan gum added was 1%, the chewiness reached the maximum value of 42.9 g, the springiness reached 0.287 g, the recovery force was 0.099 g, and the gumminess was 93.5 g. We found that the addition of xanthan gum made the potato starch gel exhibit higher springiness values, which was similar to the results of Chen et al. [31]. The results showed that adding an appropriate amount of xanthan gum to the basic material could enhance the interactions between potato starch polysaccharide molecular chains. The addition of a larger amount of xanthan gum was found to destroy the network structure, which is also consistent with the findings of Mudgil et al. [32], and the same trend was found in the viscoelasticity of different materials measured using rheology. In addition, Da Silva Costa et al. [20] also found that the addition of hydrocolloids could be used as an alternative method of increasing the stability and textural properties of starch gel varieties.

### 3.3. 3D Printing Performance Analysis

**Figure 3:**
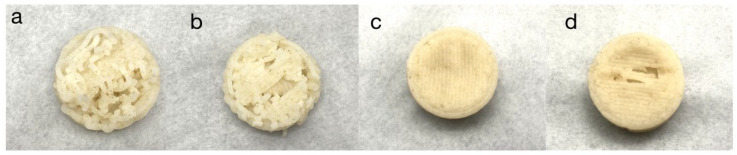
3D printing performance of materials containing (**a**) 0%; (**b**) 0.5%; (**c**) 1%; (**d**) 1.5% xanthan gum.

Three-dimensional printing stability is an important indicator used to evaluate the quality of 3D-printed products [33], and the addition of a moderate amount of xanthan gum is a way of increasing the stability and enhancing the texture properties of starch gels [20]. The 3D printed samples with 0%, 0.5%, 1%, and 1.5% xanthan gum added to the material are shown from left to right, respectively, in Figure 3. It was observed that the printed samples without xanthan gum were not properly formed, the internal lines of the solid cylinder were disordered, and the material continued to have faults and discontinuous extrusions during the extrusion process. When 0.5% xanthan gum was added to the 3D printing, the sample was still not properly formed, and the internal structure of the solid cylinder could be seen as a linear structure but was slightly better than when no xanthan gum was added. However, there were occasional fractures and extrusions that were not smooth during the extrusion process of the material. When 1% xanthan gum was added for 3D printing, the sample was in good shape, the material did not collapse after a period of observation, the overall strength was good, and the material was continuously discharged without faults during 3D printing. When 3D printing was performed after adding 1.5% xanthan gum, the overall appearance of the cylinder was slightly worse than that of the sample to which 1% xanthan gum was added. The printing process was continuous, and the support effect was slightly worse than in the sample to which 1% xanthan gum was added. Based on the comprehensive analysis of rheology, texture, and 3D printing properties during the follow-up experiments, the addition of 1% xanthan gum was selected to be used as the medium to culture *Pleurotus eryngii* mycelia.

**Figure 4:**
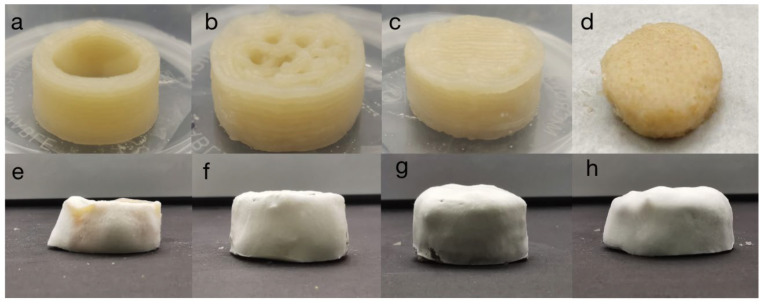
3D printing materials with filling ratios of (**a**) 0%; (**b**) 50%; (**c**) 100%; (**d**) solid medium prepared using the mold; materials covered with mycelium in (**e**) 3D-0%; (**f**) 3D-50%; (**g**) 3D-100%; (**h**) mold.

As shown in Figure 4, the images (a–d) show the samples 3D printed using the optimized medium with different filling ratios, while images (e–g) show the materials covered with mycelia after being cultured for a period of time by adding the *Pleurotus eryngii* fermentation broth. It was observed that when the filling ratio of the 3D printing setting was 0%, the mycelia grew sparsely, while some of the medium did not grow mycelia. Figure 4f shows that when the filling ratio set during 3D printing was 50%, the mycelia grew more evenly. When the filling ratio was set to 100%, the mycelia in the sample grew more vigorously than in 4f, as shown in Figure 4g. Figure 4d was the sample obtained by pressing the mold after the mycelia were overgrown. Figure 4h shows that the sample was uneven during the mold post-cultivation process.

### 3.4. Scanning Electron Microscopy

**Figure 5:**
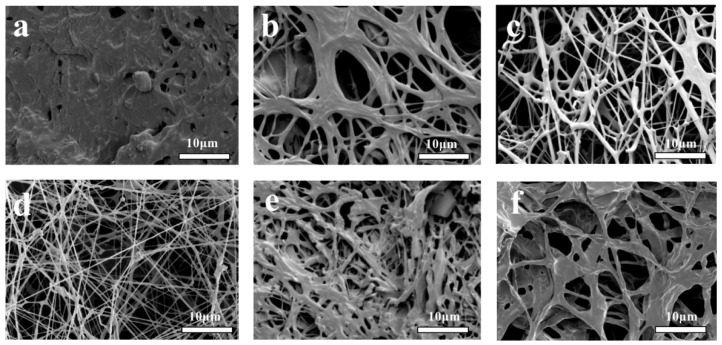
SEM images of (**a**) solid medium; (**b**) mycelial growth in the solid medium for 3D-0%; (**c**) 3D-50%; (**d**) 3D-100%; (**e**) mold preparation; (**f**) liquid cultured *Pleurotus eryngii* mycelia.

Figure 5 shows the microstructure of each sample observed under a scanning electron microscope at a magnification of 5000 times. Figure 5a shows the SEM image of the culture medium, while Figure 5b–d show the SEM images of the 3D printing culture system with different filling ratios of 0%, 50%, and 100%, respectively, and the SEM images of the mold group and *Pleurotus eryngii* are shown in Figure 5e,f, respectively.

It was observed under a scanning electron microscope that the surface of the samples in the solid medium shown in Figure 5a was uneven and rough with some irregular holes, and it had a compact texture.

Figure 5b–d shows the growth of the mycelia on the 3D-printed culture system and it was observed that the increase in the 3D printing filling ratio, increased the density of the mycelia. In the sample with a filling ratio of 0%, a large area of the medium was observed. The connection between the mycelia and the solid medium formed a dense network structure, and the distribution of the mycelia on the medium was relatively scattered and the number of mycelia was lower. In samples with a filling ratio of 50%, the growth state of the mycelia on the medium was better, and there were no fractures, while the number of mycelia was more than in samples with a filling ratio of 0% but less than in that with a filling ratio of 100%. In the samples with a filling ratio of 100%, the mycelia were very evenly distributed, the medium was not visible, the mycelia were dense, and the number of mycelia was high.

The samples of the mold group shown in Figure 5e were obtained by manual extrusion through a cylindrical mold. It was observed that the surface of the sample was rough, and there were a large number of irregular holes in the interior of the medium. The distribution of the mycelia in the medium was not uniform, and there were many breakages.

The mycelia of *Pleurotus eryngii* shown in Figure 5f were manually stacked and compressed during sample preparation. The surface structure of the large mycelia was relatively rough, there were large holes, and the mycelia was thicker when they were gathered together.

### 3.5. Starch and β-Glucan Assay

Enzymatic hydrolysis was used to increase the starch content of the solid medium to 86.21%, which was because the materials contained potato whole flour, potato starch, wheat bran and contained some starch. As the 3D printing filling ratio increased, the starch content of the samples in the 3D printing culture system showed a gradually decreasing trend, and the content decreased from 84.18% to 60.35%, which may have been because the number of mycelia in the products of 3D printing culture system gradually increased along with the increase in the 3D printing filling ratio. As it grows, the mycelia absorb nutrients from the solid medium and consume some of the high-calorie starch, resulting in a decrease in the overall starch content of the material. The starch content in the solid culture products prepared using the mold reached 67.74%, which was slightly lower than that of the 3D printing system with a filling ratio of 100%. This was inseparable from the growth state between the solid medium and *Pleurotus eryngii* mycelia. In addition, the starch content of the mycelia of *Pleurotus eryngii* was very low at 1.8%.

The Congo red method was used to determine that the β-glucan content in the solid medium was as low as 2.68%, mostly due to the addition of a small amount of wheat bran to the solid medium. In the 3D printing samples, the increase in the filling ratio of 3D printing increased the β-glucan content in the 3D printing culture system, with the highest β-glucan content of 24.31% in the sample with a 100% filling ratio. The β-glucan content in the mold group reached 22.17%, and the β-glucan in the mycelia of *Pleurotus eryngii* was richer, reaching 28.52%, which may have been due to the addition of wheat bran, while the exogenously added fermentation broth of *Pleurotus eryngii* may have also contained a small amount of β-glucan.

**Figure 6:**
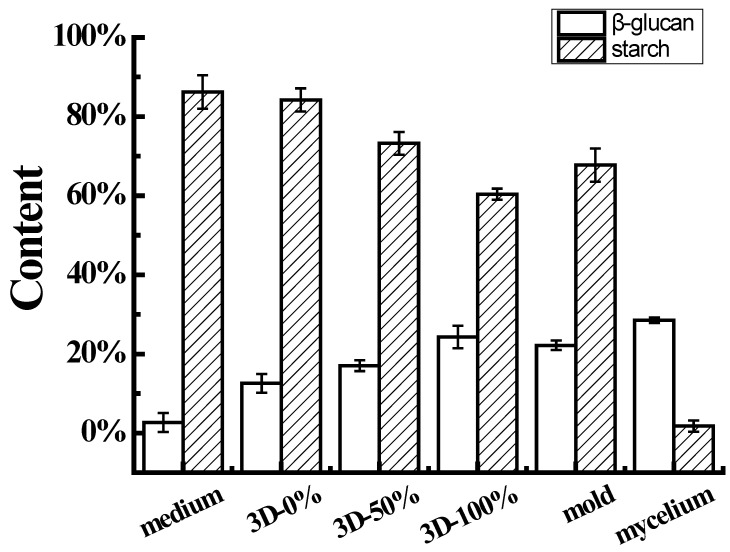
Comparison of starch and β-glucan content in different samples.

Figure 6 shows that the content of high-calorie starch and low-calorie β-glucan in the material had an opposite trend during the growth process. The content of β-glucan gradually increased while the content of starch gradually decreased. There may have been some biotransformation between starch and β-glucan. Generally, fungal glucoamylase, as well as fungal α-amylase, may be used to convert potato starch to glucose. The mycelium absorbs and uses glucose to synthesize uridine diphosphate glucose. Biosynthesis of beta-glucans in fungi involves several reactions: initiation, chain elongation, and branching, of which the most studied one is the elongation step. This reaction, catalyzed by the so-called glucan synthetases, utilizes uridine diphosphate glucose as a sugar donor [34].

### 3.6. Amino Acid Analysis

**Figure 7:**
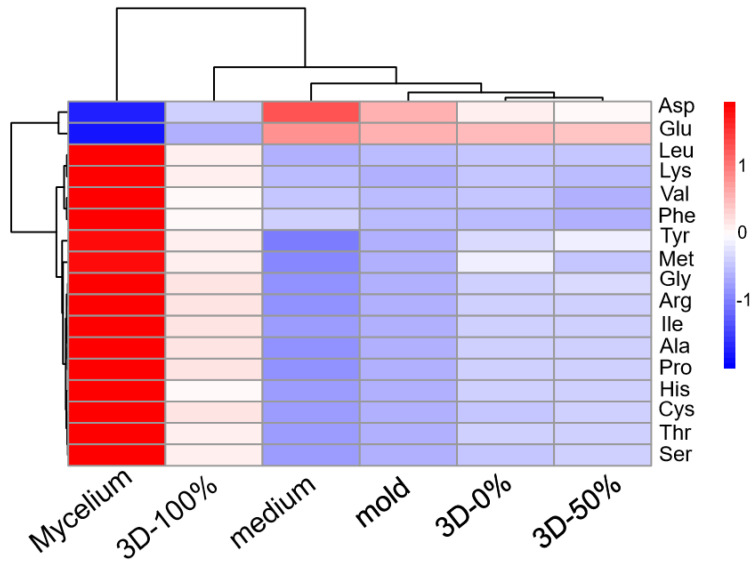
Comparison of amino acid content in the different samples.

Amino acids are essential nutrients for human survival and play an important role as constituents of tissue structure and in regulating the body’s metabolic activities. Mushrooms are a great supplementary source of essential amino acids [35]. A wide variety of amino acids have been found in samples of high-performance liquid chromatography. Figure 7 shows that the contents of the tyrosine, methionine, glycine, arginine, isoleucine, alanine, proline, histidine, cysteine, threonine, and serine within the solid medium were low, but the contents of aspartic acid and glutamic acid were higher, reaching 4.55 and 5.53 mg/g, respectively. In the 3D printing culture system, the contents of aspartic acid and glutamic acid in the samples with the filling ratio of 0% and 50% were slightly lower than those of the solid medium, and the contents of other types of amino acids were slightly higher. The amino acid content of the solid culture products prepared using the mold was similar to that of the samples with filling ratios of 0% and 50% in the 3D printing system. The detection of *Pleurotus eryngii* mycelia showed that the content of amino acids except aspartic acid and glutamic acid was higher than that of the solid medium, the 3D printing system, and the mold group, while the content of aspartic acid and glutamic acid showed low values of 2.20 and 4.94 mg/g, respectively. This may have been because the increase in the 3D printing filling ratio caused an increase in the number of pores inside the medium, and the number of mycelia grown in the medium after adding the fermentation broth also increased along with the increase in the 3D printing filling ratio. At the highest level of porosity, the mycelia absorbed nutrients provided by the solid medium to maximize nutrient exchange between the mycelia and the medium, resulting in changes in the amino acid content between the medium and the mycelia. When the filling ratio was 0% and 50%, there were fewer pores in the medium, and the mycelia grown were uneven. In addition, since the mold group was formed using manual pressing, the internal structure of the medium was relatively thick, and was not as good as that of the 3D printing system, as it had layers and gaps. The density of the mycelia growth was also less than that of products with a filling ratio of 100% in the 3D printing culture system, and the inhomogeneity of mycelia growth resulted in less transformation of nutrients between the mycelia and the medium in the mold group.

## 4. Conclusions

This study explored the role of *Pleurotus eryngii* mycelia in the transformation of high-calorie potato starch to low-calorie β-glucan. High-calorie potato starch can be used to produce edible fungal mycelium. The 3D printing culture system promoted the bioconversion efficiency of the mycelia. The potato-based ink printed using 3D technology provided a good space for the growth of *Pleurotus eryngii* mycelia. *Pleurotus eryngii* mycelia are rich in β-glucans and proteins, which can be used as a potential source of healthy food. This also shows the good application potential of the *Pleurotus eryngii* mycelia for the preparation of low-calorie food.

## Figures and Tables

**Table 1:** Gradient elution mode.

Time (min)	Flow Rate (mL/min)	Mobile Phase A (%)	Mobile Phase B (%)
0	1	92	8
27.5	1	40	60
31.5	1.5	0	100
32	1.5	0	100
34	1	0	100
35.5	1	92	8

**Table 2:** Texture characteristics of the potato powder supplemented with different amounts of xanthan gum.

XG Content (%)	Hardness	Chewiness	Springiness	Resilience	Gumminess
0	267.9 ± 47.1	13.1 ± 3.1	0.187 ± 0.013	0.077 ± 0.001	62.8 ± 18.3
0.5	223.6 ± 56.8	30.1 ± 4.7	0.224 ± 0.015	0.086 ± 0.005	78.3 ± 13.1
1	151.8 ± 39.3	42.9 ± 2.4	0.287 ± 0.005	0.099 ± 0.003	93.5 ± 7.5
1.5	230.4 ± 23.4	38.5 ± 3.4	0.231 ± 0.013	0.012 ± 0.006	47.2 ± 7.1
